# Personalized identification of tumor-associated immunogenic neoepitopes in hepatocellular carcinoma in complete remission after sorafenib treatment

**DOI:** 10.18632/oncotarget.26247

**Published:** 2018-10-23

**Authors:** Sindy Vrecko, David Guenat, Patricia Mercier-Letondal, Hugues Faucheu, Magalie Dosset, Bernard Royer, Jeanne Galaine, Romain Boidot, Stefano Kim, Marine Jary, Olivier Adotévi, Christophe Borg, Yann Godet

**Affiliations:** ^1^ University Bourgogne Franche-Comté, INSERM, EFS BFC, UMR1098, Interactions Hôte-Greffon-Tumeur/Ingénierie Cellulaire et Génique, Besançon F-25000, France; ^2^ University Bourgogne Franche-Comté, LabEx LipSTIC ANR-11-LABX-0021, Besançon F-25000, France; ^3^ University Hospital of Besançon, Department of Molecular and Cell Biology, Besançon F-25000, France; ^4^ Stanford Cancer Institute, Department of Medicine, Division of Oncology, Stanford University, Stanford, CA 94305, USA; ^5^ University Hospital of Besançon, Department of Pharmacology, Besançon F-25000, France; ^6^ Centre Georges-François Leclerc, Platform of Transfer in Cancer Biology, Department of Biology and Pathology of Tumours, Centre de Recherche INSERM LNC-UMR123, Dijon F-21000, France; ^7^ University Hospital of Besançon, Department of Medical Oncology, Besançon F-25000, France

**Keywords:** neoepitopes, mutations, hepatocellular carcinoma, CD4 T cells, sorafenib

## Abstract

Sorafenib, a multi-targeted kinase inhibitor, is the current standard systemic treatment for advanced hepatocellular carcinoma. Sorafenib has anti-angiogenic and anti-proliferative properties and is also known to favor anti-tumor T cell responses by reducing the population of immunosuppressive cells such as Treg and MDSC. Anti-tumor immune responses, especially mediated by CD4+ T-cells, are critical for tumor cells eradication and therapies modulating those responses are appealing in a growing number of cancers.

Here, we report and investigate the case of a patient diagnosed with an advanced HCC treated by sorafenib who experienced a complete histological response. We aimed to identify immunogenic peptides derived from tumor mutated proteins that stimulated CD4+ T cells responses thus favoring the exceptional recovery process of this patient.

Tumor neoantigens were identified using whole exome sequencing of normal and tumor tissue and peptide MHC binding prediction algorithms. Among 442 tumor-specific somatic variants, 50 missense mutations and 20 neoepitopes predicted to bind MHC-II were identified. Candidate neoepitopes immunogenicity was assessed by IFN-γ ELISpot after culture of patient’s PBMCs in presence of synthetic neopeptides.

CD4+ memory T cell responses were detected against a mutated IL-1β^S230F^ peptide and two additional neoepitopes from HELZ2^V241M^ and MLL2^A4458V^ suggesting that efficient anti-tumor immune response occurred in this patient. These results showed that T cells can recognize neoantigens and may lead to the cancer elimination after immunomodulation in the tumor-microenvironment induced by sorafenib. This observation indicates that other immunotherapies in combination with sorafenib could potentially increase the response rate in HCC at advanced stage.

## INTRODUCTION

Hepatocellular carcinoma (HCC), the most common primary malignant neoplasm of the liver (85%–90%) [1], is the sixth most frequent cancer in the world and the third cause of cancer-related death [2]. In the majority of patients, the disease is diagnosed at advanced stages and less than 20% of patients with HCC are eligible for curative treatments. To date, only 3 therapeutic approaches are considered as curative: surgical resection, liver transplantation and percutaneous radiofrequency ablation.

Conventional chemotherapies did not show any significant benefits in the treatment of HCC except for transarterial chemoembolization which allows a slight increase of life expectancy. In advanced stage, sorafenib has been approved as a standard, according to the Barcelona Clinic Liver Cancer (BCLC) staging and its updates [3]. In the Sorafenib HCC Assessment Randomized Protocol (SHARP) phase III trial, patients with advanced HCC were treated with sorafenib or placebo. The median overall survival significantly increased in the sorafenib group compared with the placebo group (10.7 vs 7.9 months, HR = 0.69; 95% CI:0.55 to 0.87; *p* < 0.001) [4]. However, there were no complete response in either group and objective responses rates remained poor and were between 2 and 3.3%.

Sorafenib is an oral multikinase inhibitor that mainly targets kinases involved in tumor cell growth and angiogenesis such as Raf kinases (CRAF, BRAF, V600E BRAF) and tyrosine kinases (FLT3, Kit, VEGFR2/3 and PDGFRB) [5]. *In vivo*, sorafenib has limited effects on HCC tumor cell proliferation [4]. Nevertheless, sorafenib has the potential to induce a complete remission in few cases (less than 1%) of advanced HCC cases [6]. Besides, sorafenib’s targets, such as c-Kit, VEGFR and FLT-3, are abundantly expressed in immune cells such as regulatory T cells (Treg) and myeloid-derived suppressive cells (MDSC) [7, 8]. Sorafenib has thus been implicated in the reduction of Treg and MDSC number and in the lowering levels of immunosuppressive cytokines [9–12]. Moreover, sorafenib has been shown to reduce the immunosuppressive burden by reducing PD-1 expression on circulating T cells [9, 10].

During the last decade, evidences of the impact of active antitumor immune response on clinical outcome of HCC patients have been described [13, 14]. CD3+ and CD8+ cell densities have been significantly associated with a low rate of recurrence and a prolonged relapse-free survival [15, 16]. Furthermore, high levels of intratumoral and peripheral blood Treg were associated with a higher alpha-fetoprotein (aFP) level, a more advanced TNM stage and a more vascularized tumor [15, 17, 18]. The progressive deficit of CD4+ T cells functionality induced by FoxP3+ regulatory T cells was also correlated with poor survival and high recurrence rates in HCC patients [13, 19, 20].

Antitumor immune response could be driven by the recognition of neoantigens somatically generated by mutations in tumor cells. Interestingly, most of the specific-neoantigen immune responses observed are mediated by CD4+ T cells [21] and several studies highlighted a critical role for neoantigen-specific CD4+ T cell responses in tumor elimination [21–23]. Indeed, Tran *et al.* demonstrated that adoptive transfer of CD4+ T cells specific of ERBB2IP mutation leads to an objective tumor response in metastatic cholangiocarcinoma.

The link between the effects of sorafenib on the immune system and its efficacy in advanced HCC remains a matter of investigations. We hypothesized that CD4+ T cell antitumor immune response targeting HCC preexists in some patients and that efficacy of immunomodulatory drugs such as sorafenib may be related to their immune status [24, 25]. To support this hypothesis, we aimed to identify in the present study the immunogenic mutations efficiently recognized by CD4+ T cells in an advanced HCC patient in complete histologic response after sorafenib treatment.

## RESULTS

### Complete histologic response induced by sorafenib

**Figure 1:**
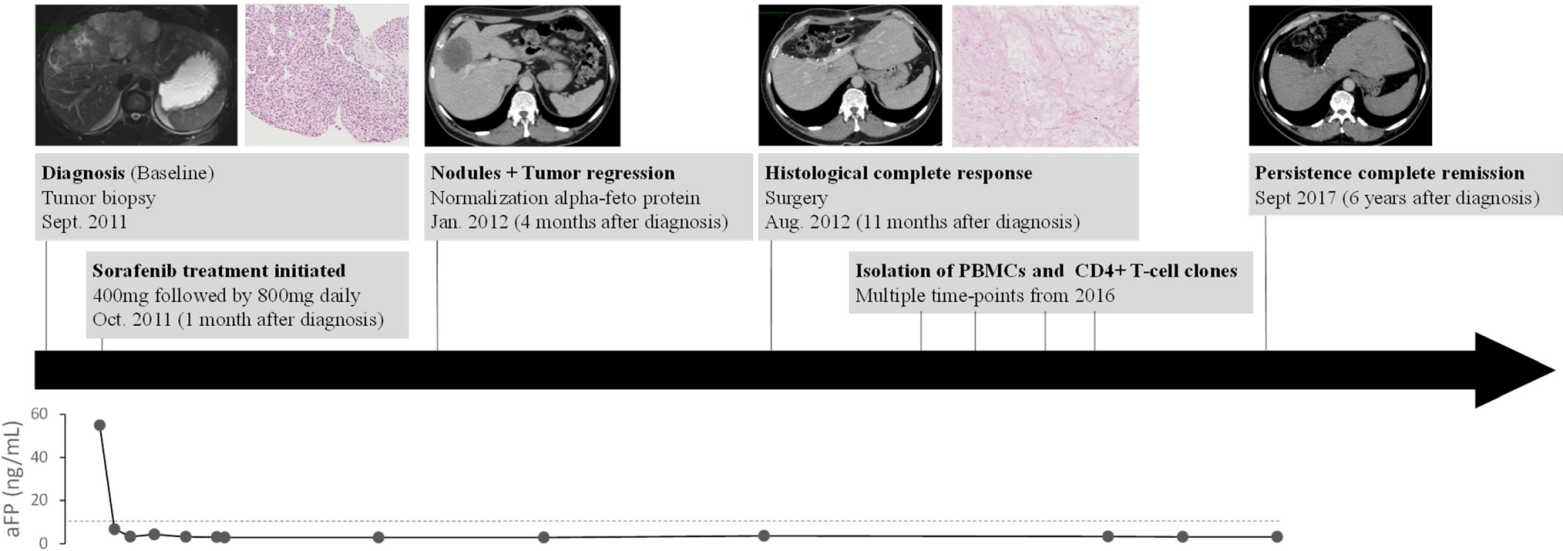
Patient’s history Timeline of diagnosis and treatment of hepatocarcinoma patient showing magnetic resonance imaging, scanner imaging and alpha fetoprotein (aFP) level at several times of pathology history.

In September 2011, a 51-year-old male patient presented with a large hypervascular liver tumor that measured 20 cm with satellite nodules disseminated in all the liver segments (Figure 1). A biopsy was performed at the University Hospital of Besançon and the pathologic examination revealed a hepatocellular carcinoma. The patient had no history of cirrhosis and extrahepatic extension assessment was negative. The patient’s serum aFP level was 55 ng/mL. In October 2011, sorafenib therapy was initiated at a dosage of 200 mg twice per day and rapidly followed by 400 mg twice per day for 8 months. No side effects were observed expect a moderate grade 1 hand foot syndrome and grade 1 diarrhea that made necessary a temporary reduction of the posology to 200 mg twice per day. After 3 months of treatment a partial response was observed, with a substantial reduction of the tumor burden from 20 to 7.5 cm. After 11 months, a complete surgical resection of the tumor area was achieved and pathologic examination revealed a complete histologic response. Five years later, the patient was still free of disease.

### Mutational profiling of the hepatocellular carcinoma

**Figure 2:**
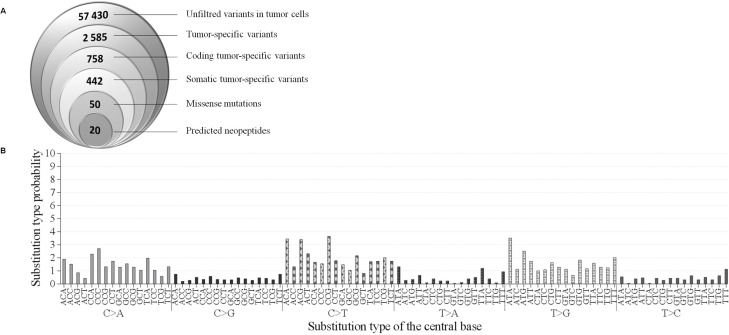
Mutations in patient’s hepatocarcinoma (**A**) Identification of suitable neoepitopes by reverse immunology. Venn diagram (from left to right): Number of mutations detected in tumor sample by WES. Number of tumor-specific variants, somatic tumor-specific variants and missense mutations found to be expressed. Finally, number of neopeptides predicted to bind to HLA-DRB1^*^15:01 with a binding score ≥20 (Syfpeithi) and/or a percentile rank <10 (Immune epitope). (**B**) Pattern of signatures of the mutational processes operative in HCC exome. The mutational signature is displayed using a 96-substitution classification defined by the substitution class and the sequence context immediately 3′ and 5′ to the mutated base.

To identify candidate immunogenic neoantigens, we applied an inverse immunological strategy. A whole exome sequencing (WES) was carried out on the tumor biopsy at diagnosis as well as on autologous normal hepatocytes from the resected liver tissue. The WES identified 57,430 unfiltered variants in cancer cells (Figure 2A). Variants were found in genes known to be mutated in HCC [26] such as SF3B1, APOB and APOBR. However these genes presented only common SNP mutations thus questioning their implication in oncogenesis. Comparison of the 57,430 variants with normal cells resulted in the identification of 2,585 variants only found in tumor cells and 758 of them had coding mutations. Among them, 442 were somatic tumor specific mutations, and 50 of these being missense mutations (Supplementary Table 1). These 50 mutations were used to establish a list of candidate neoepitopes which could bind patient’s MHC-II alleles.

The number of somatic mutations in this patient is in line with the somatic mutation load commonly observed in HCC [27], which is not belonging to the hypermutated cancers group [28]. Among proteins encoded by the 50 missense tumor specific mutated genes, some are reported to be frequently mutated in other cancers in the COSMIC database (accessed in June 2017). For example, MUC16 was found mutated in 7.4% of cancers (2225 mutated out of 30047 tested samples), MLL2 in 4.8% (1633 out of 34019), FAT1 in 3.81% (1169 out of 30688) and BAI3 in 2.75% (823 out of 30208) (Supplementary Table 1). In addition, with the exception of SYK, no clear oncogenic kinase targeted by sorafenib was identified as mutated. Among 50 SNV mutations studied, only three have already been reported in COSMIC database: ANKRD42R119Q, CLMNK488E, FASNR425W, respectively in an endometria carcinoma, skin carcinoma and stomach carcinoma (COSMIC database, 2017-June).

Mutational signature of single-nucleotide variants (SNV) was also studied by analyzing the distribution of each mutation in 96-trinucleotide combinations as described by Alexandrov *et al.* [27]. Each combination is defined by the substitution class (C>A; C>G; C>T; T>A; T>C; T>G) and the sequence context immediately 5′ and 3′ to the mutated base (Figure 2B). A mutational signature which does not seem to correspond to any described mutation signature in the COSMIC database (http://cancer.sanger.ac.uk/cosmic/signatures) was identified [27]. This observation suggests a mutational signature with a predominance of (C>A), (C>T) and (T>G) substitutions at trinucleotide motifs in cancer cells, without exceeding 4% of total SNV.

### *In silico* prediction of tumor-specific neoepitopes

MHC-II genotyping indicated that the patient is homozygote for HLA-DRB1^*^1501, HLA-DPB1^*^0401 and HLA-DQB1^*^06. As the magnitude of the HLA-DR-restricted responses have been described as significantly higher than HLA-DP [29], we focused on HLA-DRB1^*^1501 to identify candidate neoepitopes. An *in silico* prediction approach was performed using both Syfpeithi and Immuneepitope algorithms with the protein sequences encoded by the 50 missense cancer-specific mutations. Twenty peptides were predicted to bind HLA-DRB1^*^1501 molecules with a binding score ≥ 20 (Syfpeithi) and/or a percentile rank < 10 (Immune epitope). The predicted IC50 (nM) of each mutant or WT peptides were reported (Table 1). Several peptides identified are predicted to also bind HLA-DPB1^*^0401, but not HLA-DQB1^*^0601 (data not shown). Some mutations are predicted to enhance the peptide affinity for HLA-DRB1^*^1501 and HLA-DPB1^*^0401 (i.e.: GABRG2^S306Y^, HHIPL1^P386L^ and IL-1β^S230F^), and others do not significantly modify their affinity (i.e.: HELZ2^V241M^, MLL2^A4802S^ and MMP3^R303S^) (Table 1).

**Table 1:** List of predicted epitopes

					Syfpeithi prediction	Immune epitope prediction			
					HLA-DRB1^*^1501			HLA-DPB1^*^0401	
	Peptide names	Gene names	Protein names	Mutated/WT peptides	Mutated/WT binding score	Mutated/WT percentile rank	Mutated/WT IC50 (nM)	Mutated/WT percentile rank	Mutated/WT IC50 (nM)
**Pool-1**	42	ANKRD42	Ankyrin repeat domain containing protein 42	TLQIML(Q/R)SGVDPSVT	24/24	11.84/11.73	637.7/605.95	67.88/66.37	9676.4/14367.5
	43	C5orf60	Putative uncharacterized protein c5orf60	QAEVGEWLRI(R/G)NKYI	30/30	2.33/2.36	27.8/43.45	54.76/63.1	5470.3/8301.65
	44	CRAMP1L	Protein crampedlike	Y(K/E)HGKDFEAIQNNIA	24/24	12.18/13.55	2184.7/2301.2	54.99/57.82	5768.8/6145.7
	45	DBC1	Cell cycle and apoptosis regulator protein 2	ISDVQVF(W/G)YSLRFNA	24/24	6.47/1.91	228.95/222.1	2.21/14.5	268.1/1198.2
	46	DCAF4L2	DDB1 and CUL4 associated factor 4 like protein 2	SLSIHAYHSFST(S/G)LS	34/34	0.80/0.83	50.85/55.45	16.5/20.02	670.45/1009.95
	47	FAT1	Protocadherin Fat 1	LNRKILYSLIDSAD(E/G)	20/20	10.06/8.41	405.6/364.9	32.52/33.85	2508.4/2655.6
**Pool-2**	48	GABRG2	Gamma-aminobutyric acid receptor subunit gamma-2	AVPART(Y/S)LGITTVLT	24/14	7.39/32.28	514.9/2106.4	10.8/57.49	1154.75/9219.3
	**49**	**HELZ2**	Helicase with zinc finger domain 2	R(M/V)QAASFGTFEQWVV	24/24	9.04/9.04	335.5/ 336.45	4.05/4.05	353.2/ 369.35
	50	HHIPL1	HHIP-like protein 1	AAQ(L/P)EVYALGVRNMW	24/14	9.46/24.22	316.7/901.25	36/52.56	5077/9003.8
	**51**	**IL1B**	Interleukin 1 beta	EFE(F/S)AQFPNWYISTS	26/18	0.62/18.71	29.55/277.35	2.67/6.5	235.6/338.9
	52	JARID2	Jumonji And AT-Rich Interaction Domain Containing 2	HKCI(C/Y)KGRSVSLTTF	24/24	11.51/6.48	865.9/327.8	53.58/35.27	12181.85/3751.35
**Pool-3**	**53**	**MLL2**	Histone-lysine N-methyltransferase 2D	(S/A)GHLLLQKLLRAKNV	20/20	2.84/2.87	116.4/117.3	15.34/14.44	688.4/659.85
	54			MV(V/A)VAELLSMKIPNS	24/24	2.28/3.75	94.95/101.05	16.21/20.76	1608.15/2029.7
	55	MMP3	Stromelysin-1	(S/R)GEILIFKDRHFWRK	20/20	0.52/0.47	25.6/24.8	10.59/10.36	591.2/586.5
	56	OR51V1	Olfactory receptor 51V1	TMAFDRYIAICNP(V/L)R	32/32	4.19/4.55	180.65/171.65	12.76/11.68	602/564.1
	57	PCDHGB7	Protocadherin gammaB7	LFLLAVILAIAL(C/R)LR	24/24	0.8/0.79	663.85/54.95	11.88/11.88	1141.95/1132.05
**Pool-4**	58	PCK1	Phosphoenolpyruvate carboxykinase, cytosolic	VARIESK(M/T)VIVTQEQ	20/20	8.49/8.49	370.55/656.2	36.51/61.89	7447.6/11284.9
	59	RHOBTB1	Rho-related BTB domain-containing protein 1	SVQPG(H/P)FRTLLQFLY	24/24	11.51/11.51	264.5/ 369.45	4.27/6.48	366.1/433.6
	60	SLC38A4	Sodium-coupled neutral amino acid transporter 4	DELLHAYS(E/K)VYTLDI	30/30	5.76/1.5	119.6/63.55	5.9/4.73	640.95/672.95
	62	PNPLA7	Patatin-like phospholipase domain-containing protein7	A(S/A)AGPLLKRSHSVPA	4/4	8.83/8.81	504.8/504.65	66.42/62.81	12199.95/11120.4

Abbreviations: WT, wild type.

Candidate peptides from mutated proteins are selected for their predicted capacity to bind HLA-DRB1^*^1501 using prediction algorithms SYFPEITHI (binding score) and Immune epitope database and binding prediction (IEDB) (percentile rank and IC50 (nM)). These 20 candidate neopeptides are divided into 4 pools, from 1 to 4. The IEDB consensus tool was also used to predict HLA-DPB1^*^0401 binding peptides.

### Identification of immunogenic tumor-associated neoepitopes

**Figure 3:**
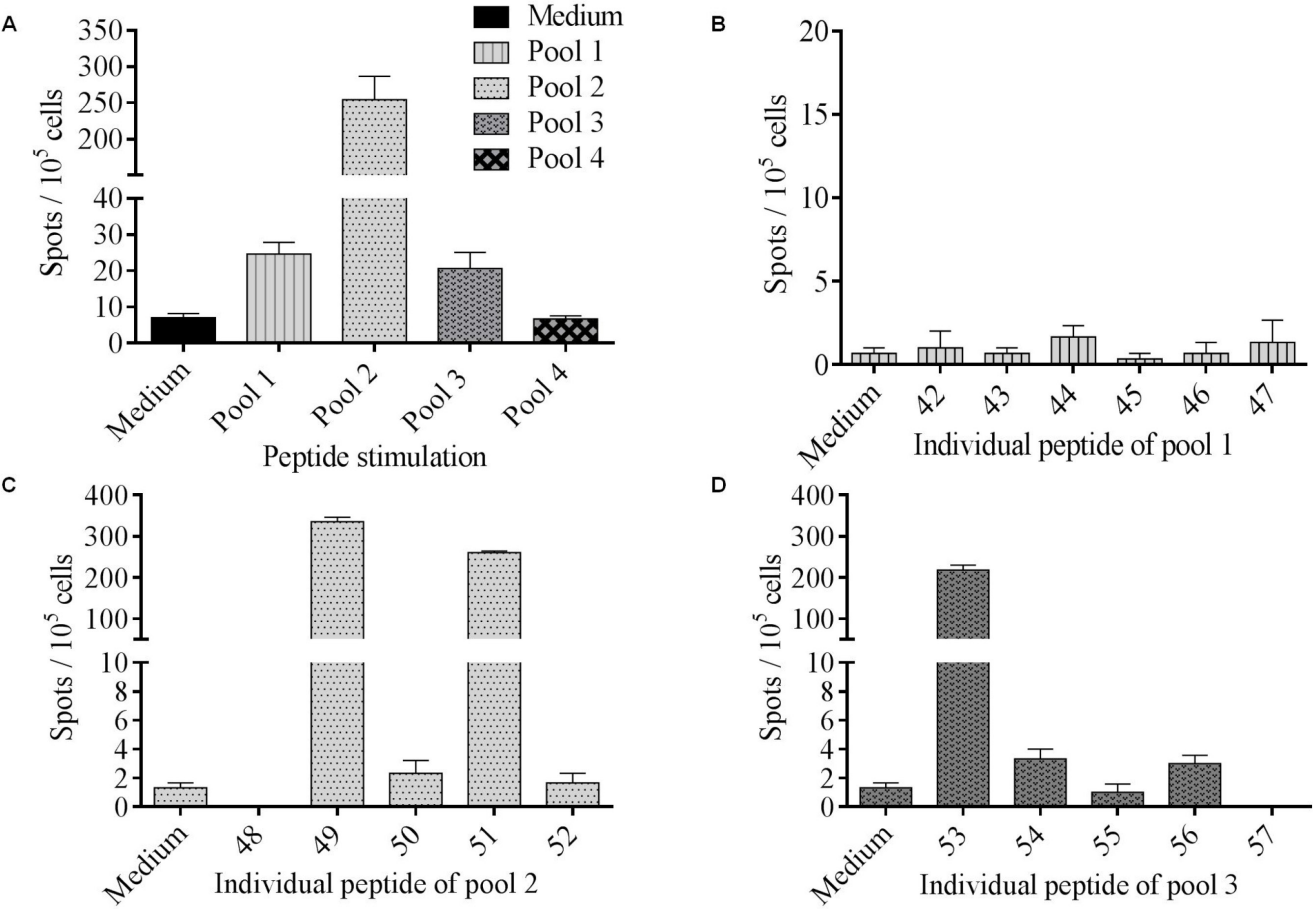
Identification of immunogenic tumor-associated neoepitopes (**A**) PBMC were cultured during 12 days with 4 pools of mutated peptides (2 μM) and the T cell reactivity was detected by IFN-γ ELISpot assay. Columns represent the mean of triplicate of IFN-γ spots number for 10^5^ cells; bars, SEM. (**B**–**D**) To assess mutated peptides-specific immune responses, PBMC were stimulated overnight with each pool separately. Neopeptides were tested individually among positive immune response inducing peptide pools: pool 1 (B), pool 2 (C) and pool 3 (D).

The ability of the selected neopeptides to stimulate CD4+ T cells was then tested. For this purpose, lymphocytes isolated from patient’s peripheral blood were stimulated *in vitro* using a pool of neopeptides (Table 1). T cells secreted IFN-γ against pool-1, -2 and -3 while no response was observed against pool-4 (Figure 3A). To identify which specific neoepitopes within neopeptide pools stimulated T-cells, we deconvoluted all stimulatory pools (Figure 3B–3D). Peptides named 49, 51, both from pool-2, and 53 from pool-3 were identified as immunogenic. No immunogenic peptides could be identified from pool-1. Thus, patient’s PBMC recognized at least three neopeptides named 49, 51 and 53 corresponding to HELZ2^V241M^ (Helicase with zinc finger domain 2), IL-1β^S230F^ (Interleukine-1β) and MLL2^A4802S^ (Histone-lysine N-methyltransferase 2D) mutations (Supplementary Table 1). Interestingly, neopeptides 51 and 53 are predicted to have a better binding affinity for HLA-DRB1^*^1501 and HLA-DPB1^*^0401 than their wild type (WT) counterpart. They are also predicted to have a better binding affinity for HLA-DRB1^*^1501 than for HLA-DPB1^*^0401. Among the neopeptides selected, based on the percentile rank, IL-1β^S230F^ (FEFAQFPNWYISTS) and MLL2^A4802S^ (SGHLLLQKLLRAKNV) mutated peptides on HLA-DRB1^*^1501 corresponded to the second and sixth better binders respectively. In contrast HELZ2^V241M^ (RMQAASFGTFEQWVV) mutated peptide corresponds to the thirteenth for HLA-DRB1^*^1501 and the third for HLA-DPB1^*^0401.

### Detection of CD4+ memory T cell responses against tumor-specific neoantigens

**Figure 4:**
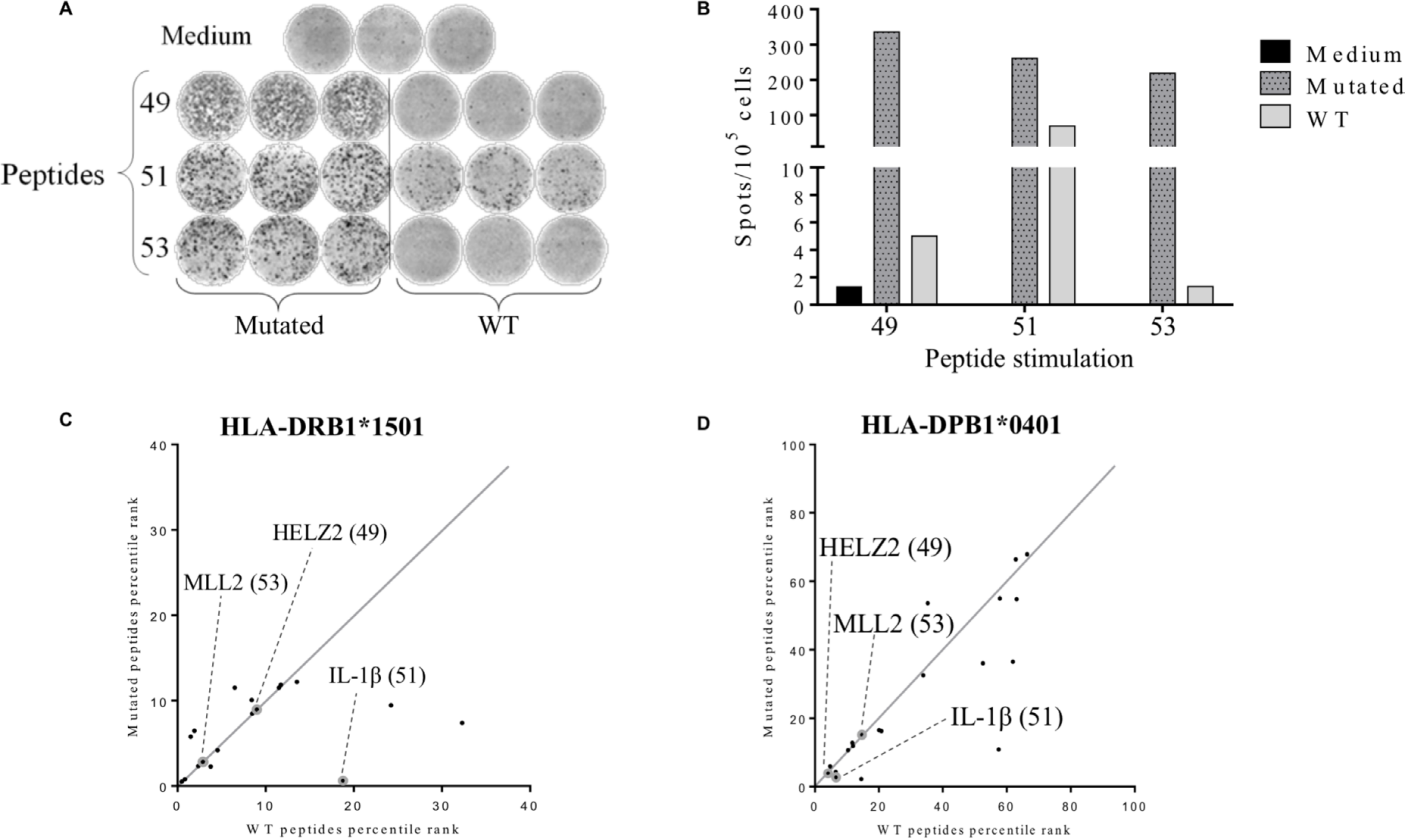
Detection of tumor-associated neopeptides-specific CD4+ T cell responses The secretion of IFN-γ in patient’s PBMCs was assessed after 12 days of stimulation by mutated or corresponding wild type (WT) peptides (2 μM) and the T cells reactivity against the peptides was detected by IFN-γ ELISpot assay as described in material and methods. Cells cultured in presence of medium were used as negative control (**A**) Illustration of medium, mutated and WT peptides IFN-γ ELISpots wells. (**B**) Histogram represents the mean of triplicate of IFN-γ spots number for 10^5^ cells. (**C–D**) Comparison between the predicted binding score of the wild type peptide with the corresponding mutated peptide for HLA-DRB1^*^1501 allele (C) and HLA-DPB1^*^0401 allele (D). Grey circles represent the identified immunogenic neopeptides: HELZ2^V241M^ (49), IL-1β^S230F^ (51) and MLL2^A4802S^ (53).

To evaluate the mutation specificity of the T cell recognition, T cells were stimulated by the mutated peptide or its WT counterpart (Table 1). Based on binding prediction IL-1β^S230F^ seemed to be a neoagretope with an IC50 of 29.55 nM for the mutated peptide versus 277.35 nM for the WT peptide in the HLA-DRB1^*^1501 context, and 235.6 nM for the mutated peptide versus 338.9 nM for the WT peptide in the HLA-DPB1^*^0401 (Table 1). However, while T cells recognized the three mutated peptides: HELZ2^V241M^, IL-1β^S230F^ and MLL2^A4802S^, only IL-1β was recognized among the WT peptides (Figure 4A and 4B). Nonetheless, IL-1β^S230F^ was able to stimulate specific immune responses with much more efficiency than the corresponding WT peptide as the number of IFN-γ secreting T cells was 3.8 times higher (Figure 4B). HELZ2^V241M^ and MLL2^A4802S^ which were not predicted to increase HLA binding affinity for HLA-DRB1^*^1501 (Figure 4C) and HLA-DPB1^*^0401 (Figure 4D) may be implicated in the TCR-HLA/peptide recognition. Overall, we demonstrated the presence of tumor-specific CD4+ memory T cell responses against 3 neopeptides, IL-1β^S230F^, HELZ2^V241M^ and MLL2^A4802S^.

### CD4+ T cell recognition of processed mutant proteins and HLA restriction

**Figure 5:**
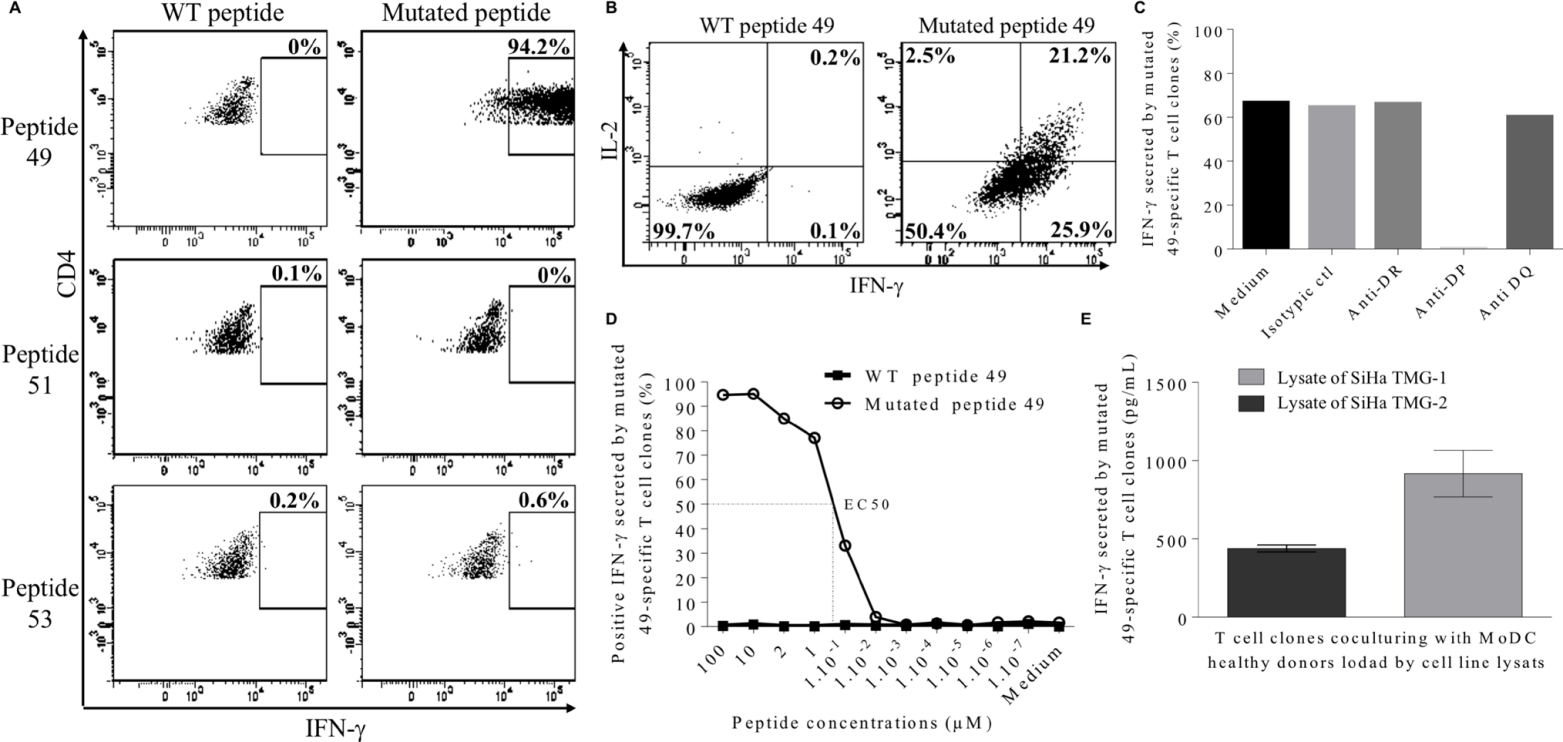
Characterization of neopeptide 49-specific CD4 T cell clones (**A**) Percentage of IFN-γ-producing CD4 T cell clone in response to 2 μM of neopeptide 49, neopeptide 51 or neopeptide 53. (**B**) Secretion of IFN-γ and IL-2-by CD4 T cell clone in response to 2 μM of HELZ2-derived WT peptide 49 (left) versus mutated peptide 49 (right). (**C**) Neopeptide 49-specific CD4 T cell clones were treated with blocking antibodies (anti- HLA-DP, anti-HLA-DR or anti-HLA-DQ) or with an isotype control before stimulation with HELZ2^V241M^ neopeptide. CD4 T cell clones reactivity was assessed by intracellular IFN-γ staining. (**D**) CD4 T cell clones were cultured in presence of B-EBV cell line loaded with increasing doses of neopeptide 49 (empty circle) and their reactivity was assessed by intracellular IFN-γ staining. Stimulation with WT peptide-loaded B-EBV is used as negative control (black triangle). (**E**) HLA-DRB1^*^04 positive allogenic MoDC loaded with tumor cell lysates from SiHa cell line transfected with TMG-1 control vector or TMG-2 vector encoding the HELZ2^V241M^ mutation were cocultured with neopeptide 49-specific CD4 T cell. Reactivity of neopeptide 49-specific CD4 T cell clones was evaluated by IFN-γ ELISA. Results are shown as mean of IFN-γ levels; bars, SEM.

To further characterize these responses, the isolation of neopeptide-specific CD4+ T-cell clones was realized after a step of IFN-γ+ cell sorting assay. Clones were successfully obtained from HELZ2^V241M^ peptide stimulated PBMCs but not from the two other T cell lines. As shown in Figure 5A, CD4+ T cell clones were only able to recognize the HELZ2^V241M^ neopeptide (49) and not the IL-1β^S230F^ (51) or MLL2^A4802S^ (53) neopeptides. In addition, we showed that the clones stimulated by HELZ2^V241M^ neopeptide mainly produced IFN-γ and IL-2, in agreement with a Th1 polarization (Figure 5B). Thus, these results showed that HELZ2^V241M^-specific CD4+ T-cell clones can be generated from the patient’s peripheral blood and were Th1 polarized. To further identify the HLA context of this recognition we co-cultured a HELZ2^V241M^-specific CD4+ T cell clone with pan HLA-DR, HLA-DP and HLA-DQ blocking antibodies. While no difference of IFN-γ secretion was found between control and pan HLA-DQ or -DR blocking antibodies, IFN-γ secretion was completely abrogated in presence of pan HLA-DP blocking antibodies (Figure 5C). The patient being HLA-DPB1^*^04 homozygous, the CD4+ T cell clone recognize the HELZ2^V241M^ peptide in the HLA-DPB1^*^04 context. Thus, these results implied that memory HELZ2^V241M^/HLA-DPB1^*^04 specific CD4+ T cells were present in patient’s blood. Finally, HELZ2^V241M^-specific CD4+ T-cell clones were stimulated by HLA-DPB1^*^04 expressing B-EBV pulsed with a range of peptide concentrations from 100 to 10^−7^ μM. The EC50 of IFN-γ secretion was observed at peptide concentration of 500 nM of HELZ2^V241M^ peptide whereas no IFN-γ was produced in response of HELZ2 WT (Figure 5D). Despite of the low T cell clone avidity, co-culture assays were realized with allogenic MoDC to assess the processing of the HELZ2^V241M^ peptide. MoDC were pulsed with cell lysate of SiHa cell line transfected with Tandem MiniGene (TMG) -1 or -2 and co-culture overnight with CD4+ T cell clones. These TMG encode a tumor immunogenic neoantigens in Sorafenib-responsive HCC protein control (TMG-1) or a protein including a 35 mer peptide encompassing HELZ2^V241M^ mutation (TMG-2). IFN-γ ELISA of these co-culture revealed that HELZ2^V241M^ peptide is efficiently processed and presented to CD4+ T-cell (Figure 5E).

## DISCUSSION

In an attempt to better understand the factors involved with the effectiveness of the sorafenib treatment in HCC, we carried out a study in a patient with an advanced HCC treated with this molecule that experienced a complete histological response. Despite a moderate overall survival improvement, sorafenib is a standard for advanced HCC, according to the Barcelona criteria. Several studies demonstrated the critical importance of tumor immunity in the effectiveness of immunotherapy in HCC [16, 17, 30–32]. Sorafenib has been shown to have immunomodulatory properties, by enhancing the activity of tumor-specific T cell and by reducing the suppressive immune cell populations such as Treg and MDSC [11, 12]. Of note, a study by Cabrera *et al.* showed that sub-pharmacologic doses of sorafenib impact subsets of T cells increasing effector T cells from patient’s HCC, while blocking Treg function [10].

The interest of combining next-generation sequencing of cancer DNA with reverse immunology to identify T cell epitopes have been highlighted in recent publications [33, 34]. It has been shown that a unique tumor neoantigen could favor the elimination of cancer cell by T cells in mouse model [35] but also in human cancers [36]. Despite the apparent low frequency of tumor-reactive T cells in gastrointestinal cancer [37] and the intermediate mutation prevalence in HCC [27, 38], the strategy reported here leads to successful isolation of neoantigen-reactive T cells from peripheral blood. WES performed on patient’s normal and cancer cells had allowed to identify tumor-specific mutations. Mutation immunogenicity analysis has been focused on missense mutations, the most studied mutation type. However, out of frame and splice site mutations may be more immunogenic [39]. As far as we know, this is the first WES performed on a sorafenib-responsive HCC patient. MUC16 which was found to have an impact on the cancer immunogenicity and has been shown to be implicated in the inactivation of NK cells and monocytes [40] was found mutated. Moreover a recent study of Balachandran *et al.* identified MUC16 neoantigens in long-term survivors of pancreatic cancer [41]. However, the MUC16 mutation expressed by the patient’s tumor cells does not seem to be presented by its HLA class II molecules as low binding capacities were predicted. In our study, several mutated genes encoding proteins of which mutated peptides are predicted to bind patient’s HLA class II were identified. Three out of 20 neopeptides were recognized by patient’s PBMC suggesting the presence of tumor-specific CD4+ T cell memory responses, potentially implicated in HCC elimination. Although the three immunogenic mutations (HELZ2^V241M^, IL-1β^S230F^ and MLL2^A4802S^) identified had never been described so far, these genes are known to be mutated in HCC [28]. MLL2 is a histone methyltransferase described as driver mutation in numerous cancer types [33, 42, 43]. Its oncogenic mechanism is unclear but it was demonstrated that mutation of MLL2 in mouse cells resulted in genomic instability [44]. HELZ2 is a protein implicated in the peroxisome activity and the proliferation of tumor cells via PPAR-δ pathway activation. IL-1β is a pro-inflammatory cytokine activating the MAP kinase pathway and was reported as a potential marker influencing HCC progression from stage III to stage IV [45]. T cell responses specific of HELZ2^V241M^ and MLL2^A4802S^ were only found against mutated peptides and not against their wild-type counterparts. These data confirmed a study by Ott *et al.* [23] which found that 86% of T cell lines were preferentially reactive against the mutated compared to the corresponding wildtype peptide.

Only HELZ2^V241M^-specific CD4 T cell clones could be isolated. This may be due to the long-term expansion required to achieve sufficient cell numbers for analysis which decreases the frequency of lower proliferative T cells [23]. Previous studies showed that HLA-DR are the most immunogenic HLA class II molecules and especially compared to HLA-DPB1^*^04 [46]. However, despite the selection of neoantigens on the basis of predicted HLA-DRB1^*^1501 binding affinity, HELZ2^V241M^-specific T cell clones were restricted by HLA-DPB1^*^0401. This may be due to the lower MHC-II binding affinity required for CD4 T activation [47] and to the MHC-II peptide binding groove structure that allows more promiscuous binding of peptides [48, 49]. This result also highlights the work that is still needed to be done to improve HLA class II binding algorithms. It is thus important to select candidate epitopes by testing several HLA-binding prediction and algorithms. Our selection process may induce a potential loss of immunogenic neopeptides, but it has permitted to drastically reduce the number of tested neopeptides and to isolate 3 immunogenic neopeptides among 20 selected candidates. This number of immunogenic neopeptides is in line with the 2-4 immunogenic peptides per patient previously found in another study by Ott *et al.*[23]. The absence of HELZ2 and MLL2 WT peptides immunogenicity, suggests that somatic mutations of these proteins might generate a neoepitope. Furthermore, the WT peptides present a similar capacity to bind HLA molecules than their mutated peptides counterpart. These results suggest an implication of the mutation in the direct interaction between the peptide and the TCR. Based on binding predictions it was unlikely to found a T cell response against IL-1β WT peptide as it presented a low affinity for HLA-DPB1^*^0401 (338.9 nM) and HLA-DRB1^*^1501 (277.35 nM). However, it was described that 8 out of 10 neoepitopes with a low affinity (>500 nM), induced a tumor rejection in tumor mouse model after immunization [50]. Moreover, if tumor material had been available, it would have been interesting to analyze neoepitope recognition by tumor infiltrating lymphocyte (TIL) as it recently highlighted a higher predicted affinity of TIL than their blood counterpart in ovarian cancer [51].

In the present study, antitumor immune responses have only been evaluated after remission and not before or during patient’s treatment. Another limitation of our work is the absence of data regarding the expression of the mutated proteins. Nonetheless, in the present study we identified memory T cell responses for all these 3 immunogenic peptides 5 years after remission, which supports the idea that these proteins were expressed by tumor cells. The neoepitopes identified may thus be implicated in the enhancement of the tumor immunogenicity [23, 35, 36]. Indeed, memory T-cell responses mostly reveal dominant epitopes [52]. However we cannot assert that HELZ2^V241M^, IL-1β^S230F^ and MLL2^A4802S^ specific CD4+ T cell responses were sufficient to induce the elimination of the whole tumor cells observed during the complete response. The correlation between neoantigens specific-T cell response and the postsurgery survival in HCC has never been studied and deserves further investigations [35, 36, 53].

The ability of T cells to target unique mutations in HCC efficiently treated by sorafenib is an additional clue to extend immunotherapies to HCC patients. This observation, along with a study by Kalathil *et al.* suggesting that T cell responses preexisting in HCC were inhibited by PD-1, reinforces the idea that analyzing the PD-1 expression on circulating HCC-specific T cells would be useful to establish new immunotherapy strategies in HCC [9]. Thus, it may also be possible to identify potential sorafenib-responsive patients by identifying the PD-1 expression in circulating T cells. Finally, these data along with the present study of a great responder patient suggest a therapeutic potential for an anti-PD-1/PD-L1 immunotherapy combined to sorafenib treatment known to decrease the immunosuppressive burden in the context of advanced HCC [9].

In conclusion, in the light of the results of recent studies on the efficacy of immunotherapies in HCC, our data from a great responder patient suggest a therapeutic potential for immune checkpoint blockade in combination with sorafenib to decrease the immunosuppressive burden and reach a higher response rate in advanced HCC [31, 54, 55].

## MATERIALS AND METHODS

### Patient

Hepatocellular carcinoma patient (HCC) was recruited through the Department of Medical Oncology of the University Hospital of Besançon (France). The patient was enrolled after the signature of informed consent, and after approval by the local ethics committee.

### DNA extraction

Tumor genomic DNA was extracted from formalin-fixed paraffin-embedded (FFPE) using QIAamp DNA FFPE Tissue Kit (Qiagen) according to the manufacturer’s instructions. Prior to DNA extraction, separate hematoxylin-eosin stained slides were reviewed by a pathologist. Normal liver tissue was isolated from post-hepatectomy fixed tissue. Tumor tissue was isolated from the initial biopsy at diagnosis and was manually macrodissected. The tumor content was 70% after macrodissection. DNA and tissue samples were collected by the biobank BB-0033-00024 “Tumorothèque Régionale de Franche-Comté”.

### Whole exome sequencing

Library preparation, capture, sequencing, and bioinformatics analysis were performed by IntegraGen, Evry, France. Genomic DNA was captured using SureSelect Human All Exon v4 + UTR - 70 Mb (Agilent) according to manufacturer’s instruction and protocols without modification except for library preparation which was performed using NEBNext Ultra kit (New England Biolabs). Pooled capture-enriched DNA samples were then sequenced by paired-end 75 bases massively parallel sequencing on HiSeq 2000 (Illumina).

### Bioinformatics analysis

Base calling was performed using the Real-Time Analysis software sequence pipeline (Illumina, RTA v1.12.4.2) with default parameters. Sequence reads were mapped to the human genome build (hg19/GRCh37) using Elandv2e (Illumina, CASAVA1.8.2) allowing multi-seed and gapped alignments. The duplicated reads were removed. CASAVA1.8.2 was used to call single-nucleotide variants (SNVs) and short insertions/deletions (max. size is 300 nt), taking into account all reads per position. SNVs and indels with Q (SNPs) < 10 and Q (Indel) < 20, or regions with low mappability (QVCutoff < 90) were filtered out. The frequency with which single base differences were expected between two unrelated haplotypes (Theta parameter) was 0.01, this frequency was set to 0.001 for indels. Variant annotation took into account data available in dbSNP (dbSNP132), the 1000 Genomes Project (phase1_release_v3.20101123), Hapmap CEU (version27), the Exome Variant Server (ESP6500SI-V2-SSA137) and from an in-house database. Genetic variation annotation was realized from IntegraGen in-house pipeline.

In order to identify the germline and somatic variants, we considered that a variant is germline if its frequency is greater than 20% in normal tissue. Then to ensure only high-confidence transitions calls, we’ve considered as tumor specific variants only bases with a frequency greater than 5% in tumor tissue and with a frequency less than 1% in normal tissue. The tumor specific transitions were selected to identify the mutation signature and we used the stratification by transition contexts into a set of characteristic patterns as described by Alexandrov *et al.* [27]. This classification of SNVs is based on six base substitutions within tri-nucleotide sequence contexts including the bases immediately at 5′ and 3′ of each mutated base. Six base substitutions (C>A, C>G, C>T, T>A, T>C, and T>G) with 16 possible combinations of neighboring bases result in 96 possible mutation types. The signature obtained was compared to signatures catalogued in the COSMIC database (2017-June).

In order to select neoantigens, others filters were applied. Thus, out of around 50000 variants issued by the variant caller (CASAVA1.8.2), there were 2585 variants from Hg19 reference in tumor cells including 758 coding variants. Among coding variants, 442 missense variants from hg19 reference in tumor cells, and finally 52 variants were annotated as somatic (ie tumor specific), corresponding to potential neoantigens.

### Epitope predictions and peptide libraries

The MHCII binding predictions were made using the Immune epitope database and binding prediction (IEDB) analysis resource Consensus tool [56, 57] and Syfpeithi [58]. A list of predicted epitopes was obtained and all mutated peptides with a percentile rank < 10 and/or a binding score ≥ 20 were synthesized by Proimmune. We also calculated the mean of IC50 values provided by IEDB from SMM align and IC50 from NN align. This value is referred as IC50 in Table 1.

### Tandem mini genes (TMG) transfected cell lines

cDNA encoding 35 amino acids (17 amino acids at each side of the mutation) were selected to design TMG. A TMG is composed of 5 cDNA sequences in tandem in an expression vector (pcDNA3.1). SiHa cell line was stably transfected with TMG (Qiagen Effectene transfection reagent kit 301425) and used to assess the specificity of neopeptide-specific clones.

### Assessment of spontaneous antigen-specific T cell response in cancer patients

Peripheral blood mononuclear cells (PBMC) were isolated by density centrifugation on Ficoll-Hyperpaque gradients (Eurobio) and plated at 2.106 cells per well in a 24-wells plate in RPMI 10% human serum with the mixture of the four pools of peptides (2 μM) as previously described. 50 Recombinant interleukins, IL-7 (5 ng/mL; Peprotech, 200–07) were added at day 1 and IL-2 (20 UI/mL; Novartis) at days 3 and 6. Specific responses were assessed at day 13 by IFN-γ ELISPOT (DIACLONE ELISpot kit 856 051 020P).

Briefly, PBMC (1.105 per well) were cultured on anti-human IFN-γ monoclonal antibody- coated ELISPOT plate with each peptide (2 μM) in *X-VIVO* 15 medium (Lonza) for 18 h at 37° C. Cells cultured with medium alone or PMA (100 ng/mL; Sigma-Aldrich) and ionomycin (10 μmol/L; Sigma-Aldrich) were used as negative and positive controls, respectively. The IFN-γ spots were revealed following the manufacturer’s instructions. Spot-forming cells were counted using the C.T.L. Immunospot system (Cellular Technology Ltd). Responses were considered as positive if spot numbers were superior to 10 and more than twice the number of background spots.

### CD4 T-cell clones isolation and amplification

Specific T-cell clones of mutated peptide 49 were sorted after IFN-γ T cell sorting according to manufacturer’s instruction after 6h of mutated peptide 49 stimulation (Miltenyi Biotec, 130-054-201). IFN-γ secreting T cells were cloned by limit dilutions and amplified after stimulation by PHA in presence of 35Gy irradiated allogeneic PBMCs and 150 UI/mL of IL-2 according to previously described procedure [59].

### Functional assessment of CD4+ T cell clones

Functional analyses of neoantigens-specific CD4+ T-cell clones were performed by using intracytoplasmic IL-2 and IFN-γ staining (ICS). Briefly, after a 14 h stimulation period with or without 2 μM, T cells were labeled with fixable viability dye (FVD) (eBioscience, 65-0865-14), anti-CD3 (BD Biosciences, 558117), anti-CD4 (Diaclone, 954.031.010), anti-IFN-γ (BD Biosciences, 554702) using Cytofix/CytoPerm KIT (BD Biosciences, 554714). Stained cells were acquired on a BD FACS Canto II (BD Biosciences) and analyzed with the BD FACS DIVA software. The HLA restriction of the specific TCR was determined with CD4+ T cell clones treated with 10μg/mL anti-HLA-DP (B7/21) (Leinco, H260) or anti-HLA-DQ (Bio-rad, MCA3796) or anti-HLA-DR (L243) (BD Biosciences, 555809) antibodies for 30 min before addition of 2 μM of neopeptides for 14 h before IFN-γ ICS. To assess the avidity of T cell clones, 1.105 T cell were culture with 1.105 peptide loaded B-EBV cells (HLA-DRB1^*^1501 and HLA-DPB1^*^0401) for 14 h before IFN-γ ICS.

### Processing of neopeptide HELZ2^V241M^ (49)

To study the processing and natural recognition of mutated peptide 49 by T cell clones, transfected-tumor cell line lysate was loaded on immature MoDC derived from healthy donors. DC were obtained from monocytes cultured for 5 days with 1000 UI/mL of IL-4 (Peprotech 200-04) and GM-CSF (Peprotech 300-03). Transfected- SiHa cell lysates (20.106 cells/ml) were loaded on immature MoDC for 24 h at 37° C. Lipopolysaccharide (LPS; Sigma, L2630) 1 μg/ml was added as a maturation signal for the last 6h of culture. After PBS 1X washing, mature loaded-MoDC were cultured with peptide 49-specific T cell clones at a 1:1 ratio for 18 h at 37° C. MoDC loaded with WT or mutated peptide 49 (2 μM) were used as negative and positive control respectively. IFN-γ secretion was assessed by ELISA (Diaclone ref 950.000.192) and acquired on a spectrophotometer (Tecan, XFluor4) according to manufacturer’s instructions.

## SUPPLEMENTARY MATERIALS TABLE





## Abbreviations

HCC: Hepatocellular Carcinoma
MoDC: Monocyte derived Dendritic Cells
PBMC: Peripheral Mononuclear Cells
SNVs: Single-Nucleotide Variants
WES: Whole Exome Sequencing
