# Supplementary material for: Personalized identification of tumor-associated immunogenic neoepitopes in hepatocellular carcinoma in complete remission after sorafenib treatment
Source: Oncotarget. 2018 Oct 23;9(83):35394–407. doi: 10.18632/oncotarget.26247 (PMC6226040; doi:10.18632/oncotarget.26247)
Supplement: Supplementary file 1 [file oncotarget-09-35394-s001.pdf]

Personalized identification of tumor-associated immunogenic neoepitopes in hepatocellular carcinoma in complete remission after sorafenib treatment

SUPPLEMENTARY MATERIALS

Supplementary Table 1: List of candidate neoepitopes: missense mutations among somatic HCC-specific mutations.
See Supplementary_Table_1
