# Supplementary material for: Personalized identification of tumor-associated immunogenic neoepitopes in hepatocellular carcinoma in complete remission after sorafenib treatment
Source: Oncotarget. 2018 Oct 23;9(83):35394–407. doi: 10.18632/oncotarget.26247 (PMC6226040; doi:10.18632/oncotarget.26247)
Supplement: Supplementary file 2 [file oncotarget-09-35394-s002.docx]

**Supplementary Table 1: List of candidate neoepitopes: missense mutations among somatic HCC-specific mutations.**

| **Gene name** | **Reference name** | **Mutation** | **WT Amino Acid sequence** | **COSMIC database** | | |
| --- | --- | --- | --- | --- | --- | --- |
|  |  |  |  | **Number of mutated/tested samples for each mutated genes** | **Percentage of each mutated genes** | **Specific mutations found** |
| ADAMTS13 | NM_139025 | G845V | GGAGLALENETCVPGADGLEAPVTE(G)PGSVDEKLPAPEPCVGMSCPPGWGH | 286 / 29588 | 0.97% | 0 |
| ADAMTS15 | NM_139055 | G661A | VDGTLCSPDSTSVCVQGKCIKAGCD(G)NLGSKKRFDKCGVCGGDNKSCKKVT | 231 / 29565 | 0.78% | 0 |
| ANKRD42 | NM_182603 | R119Q | QDDRGCTPLHLAATHGHSFTLQIML(R)SGVDPSVTDKREWRPVHYAAFHGRL | 79 / 29380 | 0.27% | 1 |
| BAI3 | NM_001704 | G86R | LDKKLKHRAGQMSEPHSGLTLKCAK(C)GVVSTTALSATTASNAMASLWSSCV | 823 / 30208 | 2.72% | 0 |
| C5orf60 | NM_001142306 | A318V | EPSSVPPREEDSENDQAEVGEWLRI(G)NKYITLKDYRILLKELENLEIYTFL | 39 / 29393 | 0.13% | 0 |
| CADPS | NM_003716 | A318V | NACQLDNPDEQAAQIRRELDGRLQM(A)DQIARERKFPKFVSKEMENMYIEELK | 399 / 29568 | 1.35% | 0 |
| CCDC79 | NM_001136505 | Q653R | EDRYKSELRKSLICNKKILLTPRRR(Q)RLSNESTTPGGIKKRRIRKNFTEEE | 87 / 29289 | 0.30% | 0 |
| CCT7 | NM_006429 | W474L | LCDNAGFDATNILNKLRARHAQGGT(W)YGVDINNEDIADNFEAFVWEPAMVR | 77 / 29379 | 0.26% | 0 |
|  |  | W474C | LCDNAGFDATNILNKLRARHAQGGT(W)YGVDINNEDIADNFEAFVWEPAMVR |  |  | 0 |
| CKAP5 | NM_001008938 | L1948M | GNTNGEEVGPSVYLERLKILRQRCG(L)DNTKQDDRPPLTSLLSKPAVPTVAS | 304 / 29380 | 1.03% | 0 |
| CLMN | NM_024734 | K488E | VLAVEVAEEKEQKQESSKIPESSSD(K)VAGDIFLVEGTNNNSQSSSCNGALE | 233 / 29468 | 0.79% | 1 |
| COL16A1 | NM_001856 | G611A | PGLPGRAGVPGLKGEKGNFGEAGPA(G)SPGPPGPVGPAGIKGAKGEPCEPCP | 312 / 29436 | 1.06% | 0 |
| CRAMP1L | NM_020825 | E180K | EGKKVRRQWESWSTEDKNTFFEGLY(E)HGKDFEAIQNNIALKYKKKGKPASM | 212 / 29290 | 0.72% | 0 |
| DBC1 | NM_014618 | G659W | GQGPVDLSDPSKRQFYIKISDVQVF(G)YSLRFNADLLRSAVQQVNQSYTQGG | 454 / 29444 | 1.54% | 0 |
| DCAF4L2 | NM_152418 | G203S | CSFQIPDAWSCAWSLSIHAYHSFST(G)LSQQVLLTNVVTGHQQSFGTSSDVL | 394 / 29384 | 1.34% | 0 |
| DCST2 | NM_144622 | M221V | DVCNSELGNPYLKCARVFDDAKDSC(M)MVIPQAYHLCYVLMPFKLALCGLAS | 157 / 29379 | 0.53% | 0 |
| EXTL3 | NM_001440 | D746Y | EKNSLNNRFLPWNEIETEAILSIDD(D)AHLRHDEIMFGFRVWREARDRIVGF | 191 / 29379 | 0.65% | 0 |
| FASN | NM_004104 | R425W | VHIILRPNTQPPPAPAPHATLPRLL(R)ASGRTPEAVQKLLEQGLRHSQDLAF | 480 / 29380 | 1.63% | 1 |
| FAT1 | NM_005245 | G3170E | TRVQATDADAGLNRKILYSLIDSAD(G)QFSINELSGIIQLEKPLDRELQAVY | 1169 / 30688 | 3.81% | 0 |
| GABRG2 | NM_198903 | S306Y | IPCTLIVVLSWVSFWINKDAVPART(S)LGITTVLTMTTLSTIARKSLPKVSY | 267 / 29668 | 0.90% | 0 |
| GLIPR1L1 | NM_152779 | M58V | FIDNCIEAHNEWRGKVNPPAADMKY(M)IWDKGLAKMAKAWANQCKFEHNDCL | 58 / 29539 | 0.20% | 0 |
| HELZ2 | NM_001037335 | V241M | PGRLYARGERFRVPSSTADFQVGVR(V)QAASFGTFEQWVVFDFGRRPVLLQK | 446 / 29383 | 1.52% | 0 |
| HHIPL1 | NM_001127258 | P386L | VDRKERGLPYGIPPDNPFVGDPAAQ(P)EVYALGVRNMWRCSFDRGDPSSGTG | 128 / 29357 | 0.44% | 0 |
| IL1B | NM_000576 | S230F | NYPKKKMEKRFVFNKIEINNKLEFE(S)AQFPNWYISTSQAENMPVFLGGTKG | 94 / 29812 | 0.32% | 0 |
| IL6R | NM_181359 | S304G | IHDAWSGLRHVVQLRAQEEFGQGEW(S)EWSPEAMGTPWTESRSPPAENEVST | 5 / 29471 | 0.02% | 0 |
| IRF4 | NM_002460 | G375S | LERDQTCKLFDTQQFLSELQAFAHH(G)RSLPRFQVTLCFGEEFPDPQRQRKL | 4 / 30857 | 0.01% | 0 |
| JARID2 | NM_004973 | Y815C | FAQEKEVVKEEEEDKGVLNDFHKCI(Y)KGRSVSLTTFYRTARNIMSMCFSKE | 290 / 29646 | 0.98% | 0 |
| KLHL8 | NM_020803 | R309L | DLLDEARNYHLHLSSRAVPDFEYSI(R)TTPRKHTAGVLFCVGGRGGSGDPFR | 124 / 29379 | 0.42% | 0 |
| KRT39 | NM_213656 | V298E | EPIMETNRKDVEQWFNTQIEELNQQ(V)VTSSQQQQCCQKEIIELRRSVNTLE | 143 / 29290 | 0.49% | 0 |
| MLL2 | NM_003482 | A4802S | REANGEPIGAPGTSNHLLLAGPRSE(A)GHLLLQKLLRAKNVQLSTGRGSEGL | 1633 / 34019 | 4.80% | 0 |
|  |  | A4458V | GKGSEVSVMLTVSAAAAKNLNGVMV(A)VAELLSMKIPNSYEVLFPESPARAG |  |  | 0 |
| MMP3 | NM_002422 | R303S | EPVPPEPGTPANCDPALSFDAVSTL(R)GEILIFKDRHFWRKSLRKLEPELHL | 145 / 29559 | 0.49% | 0 |
| MUC16 | NM_024690 | T2713S | MSETSNGDALVLKTVSNPDRSIPGI(T)IQGVTESPLHPSSTSPSKIVAPRNT | 2225 / 30047 | 7.41% | 0 |
| NIN | NM_182944 | E1243D | SEKKQDLLFDVSVLKKKLKMLERIP(E)ASPKYKLLYEDVSRENDCLQEELRM | 298 / 29857 | 1.00% | 0 |
| OR51V1 | NM_001004760 | L139V | HGLSFMESSVLLTMAFDRYIAICNP(L)RYSSILTNSRIIKIGLTIIGRSFFF | 167 / 29357 | 0.57% | 0 |
| OR6N2 | NM_001005278 | S291N | YSLTLDRTLAIVYSVLTPMVNPIIY(S)LRNKEIIKAIKRTIFQKGDKASLAHL | 189 / 29357 | 0.64% | 0 |
| PCDHGB4 | NM_032098 | G378R | LIMEDAELGTHIALLKVRDKDSRHN(G)EVTCKLEGDVPFKILTSSRNTYKLV | 293 / 29289 | 1.00% | 0 |
| PCDHGB7 | NM_018927 | R714C | QFYLVVALALISVLFLLAVILAIAL(R)LRQSFSPTAGDCFESVLCSKSGPVG | 269 / 29313 | 0.92% | 0 |
| PCK1 | NM_002591 | T92M | RRLKKYDNCWLALTDPRDVARIESK(T)VIVTQEQRDTVPIPKTGLSQLGRWM | 247 / 29473 | 0.84% | 0 |
| PRUNE2 | NM_015225 | Q2587P | KPETCEERESIAELELYVGSKETGL(Q)GTQLASFPDTCQPASLNERKGLSAE | 90 / 29424 | 0.31% | 0 |
| RAP1GAP2 | NM_015085 | E366K | PFTDGDAQQLQRKRHIGNDIVAIIFQ(E)ENTPFVPDMIASNFLHAYIVVQVET | 149 / 29290 | 0.51% | 0 |
| RGS3 | NM_144489 | E119K | LSLSLPIFPGWMEWLSPDIALPRRD(E)WTQTSPARKRITHAKVQGAGQLRLS | 216 / 29472 | 0.73% | 0 |
| RHOBTB1 | NM_014836 | P410H | MQVNPISKRMGPMTVVRMDASVQPG(P)FRTLLQFLYTGQLDEKEKDLVGLAQ | 162 / 29472 | 0.55% | 0 |
| SLC38A4 | NM_001143824 | K404E | YLLAALFGYLTFYGEVEDELLHAYS(K)VYTLDIPLLMVRLAVLVAVTLTVPI | 175 / 29379 | 0.60% | 0 |
| SYK | NM_003177 | D410Y | VKKGYYQMKKVVKTVAVKILKNEAN(D)PALKDELLAEANVMQQLDNPYIVRM | 202 / 30847 | 0.65% | 0 |
| TIA1 | NM_022173 | R69Q | GNDPYCFVEFHEHRHAAAALAAMNG(R)KIMGKEVKVNWATTPSSQKKDTSSS | 72 / 29380 | 0.25% | 0 |
| TNRC18 | NM_001080495 | L123M | TPSNLPMVQLWAAHAHEGFSHLPSG(L)YPSYLHLNHLEPPSSGSPLLSQLGQ | 422 / 29289 | 1.44% | 0 |
| TRPV5 | NM_019841 | S719G | ASLALPTSSLSRTASQSSSHRGWEILRQNTLGHLNLGLNL(S)EGDGEEVYHF | 327 / 29469 | 1.11% | 0 |
| ZNF408 | NM_024741 | P340L | QCPPRAKTPEPGAQQSGFPTLSRSP(P)GPAGSSPKQGRRYRCGECGKAFLQL | 156 / 29467 | 0.53% | 0 |
| PNPLA7 | NM_152286 | A345S | EEERLKKPPRLQESCDSDHGGGRPA(A)AGPLLKRSHSVPAPSIRKQILEELE | 249 / 29296 | 0.85% | 0 |
| SUN1 | NM_001171944 | E679Q | LRDLQLQILRNVTHHVSVTKQLPTS(E)AVVSAVSEAGASGITEAQARAIVNS | 182 / 30732 | 0.59% | 0 |
| GALNT15 | NM_054110 | W204C | PLCLQQHPQDSLPTASVILCFHDEA(W)STLLRTVHSILDTVPRAFLKEIILV | 228 / 30732 | 0.74% | 0 |

**Abbreviations:** COSMIC, Catalogue of Somatic Mutations in Cancer.

The WES carried out on the tumor biopsy and on autologous normal hepatocytes identified 57,430 unfiltered variants in cancer cells. Among them, only 2,585 variants were found in tumor cells and 758 of them had coding mutations. Among coding mutations, 442 were somatic tumor specific mutations, and 50 of these being missense mutations. These 50 missense mutations were used to establish a list of candidate neoepitopes.
